# Supplementary material for: Key Residues Affecting Binding Affinity of Sirex noctilio Fabricius Odorant-Binding Protein (SnocOBP9) to Aggregation Pheromone
Source: Int J Mol Sci. 2022 Jul 30;23(15):8456. doi: 10.3390/ijms23158456 (PMC9369295; doi:10.3390/ijms23158456)
Supplement: Supplementary file 1 [file ijms-23-08456-s001.zip › ijms-1839358-supplementary.pdf]

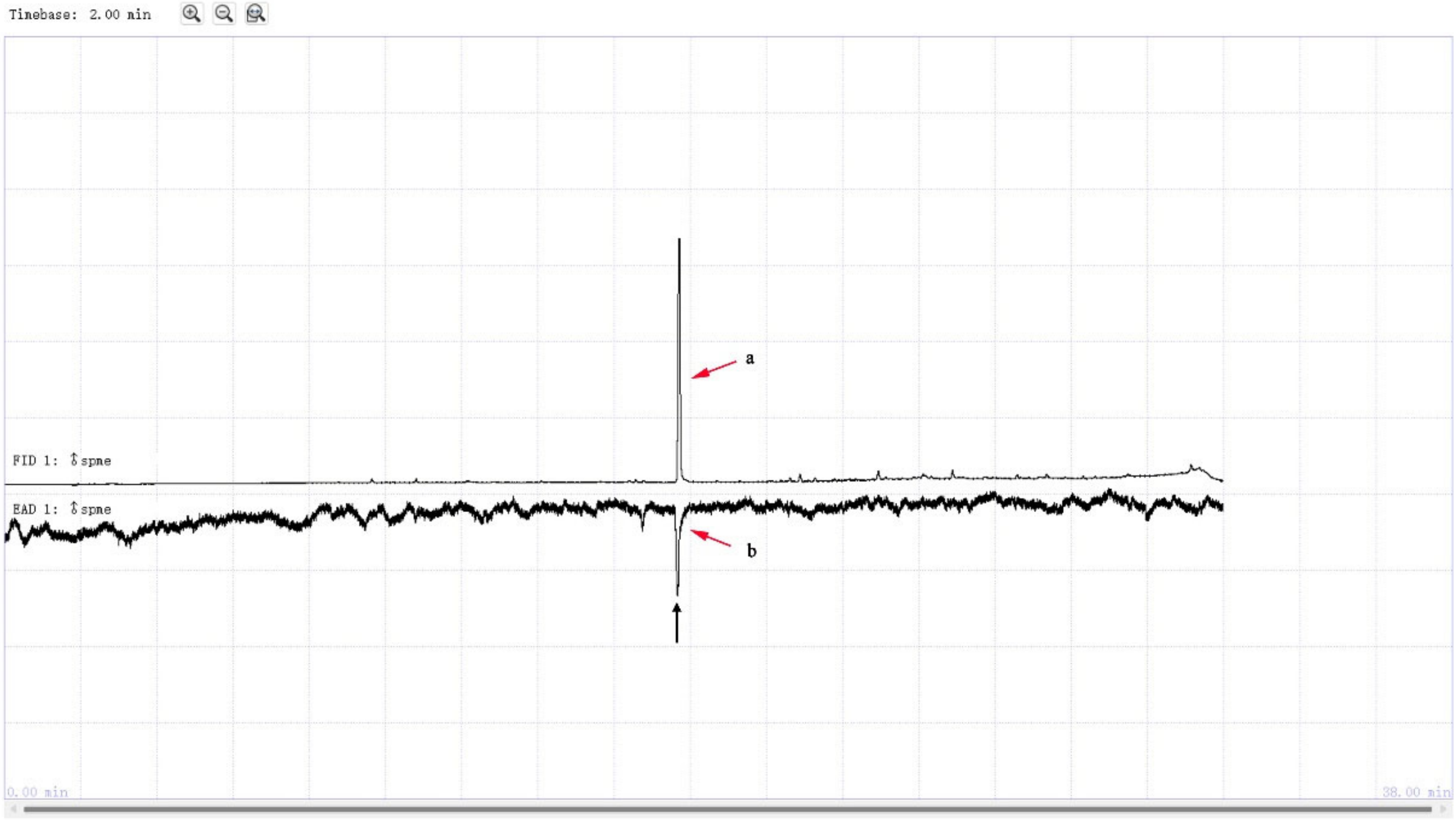

Figure S1. GC-EAD of *Sirex noctilio* to the insect aggregation pheromones released by male adults after SPME. a: (*Z*)-3-decen-ol; b: Male-consistent antennal response.

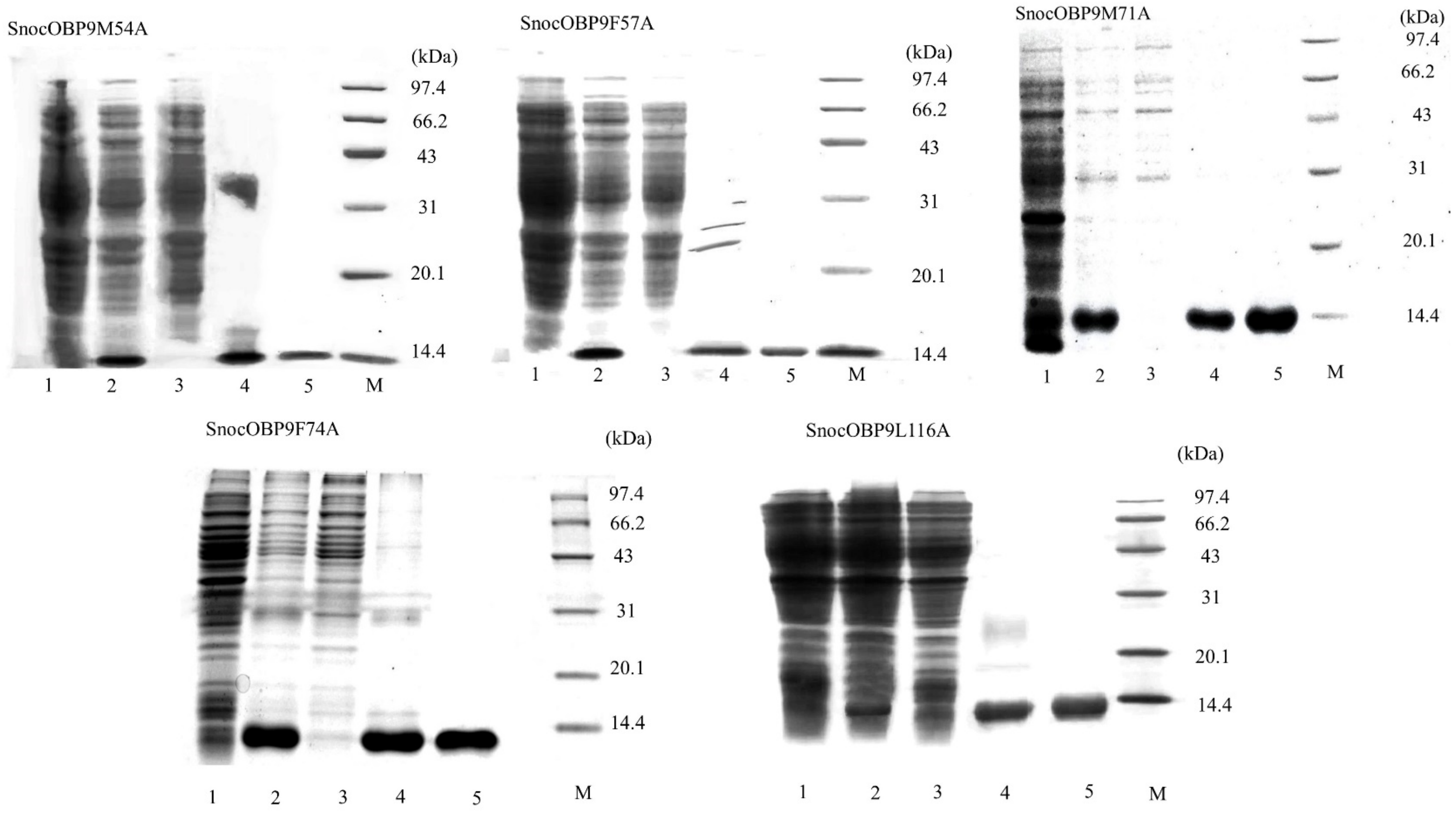

Figure S2. SDS-PAGE analyses of the expression and purification of recombinant SnocOBP9-MT. Lane 1 repre-sented the bacterial cells before inducing by IPTG; lane 2 represented the bacterial cells induced by IPTG; lane 3 represented the supernatant of the bacterial cells; lane 4 represented the inclusion body of the bacterial cells; lane 5 represented purified recombinant protein; lane M represented mo-lecular marker

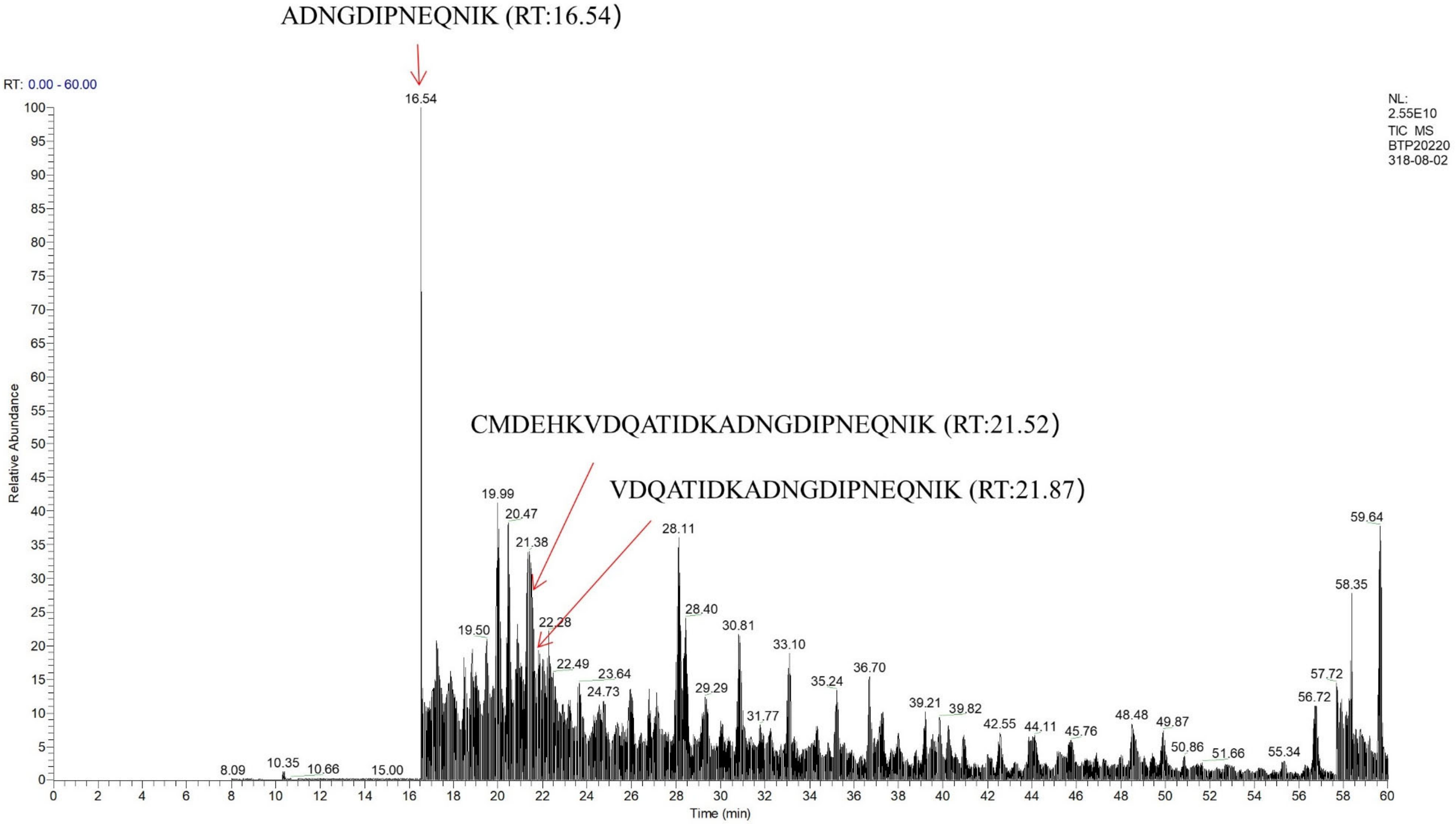

Figure S3. Total Ion Chromatogram of recombinant SnocOBP9 sample. The abscissa represents the scan time, and the ordinate represents the relative abundance of ions.

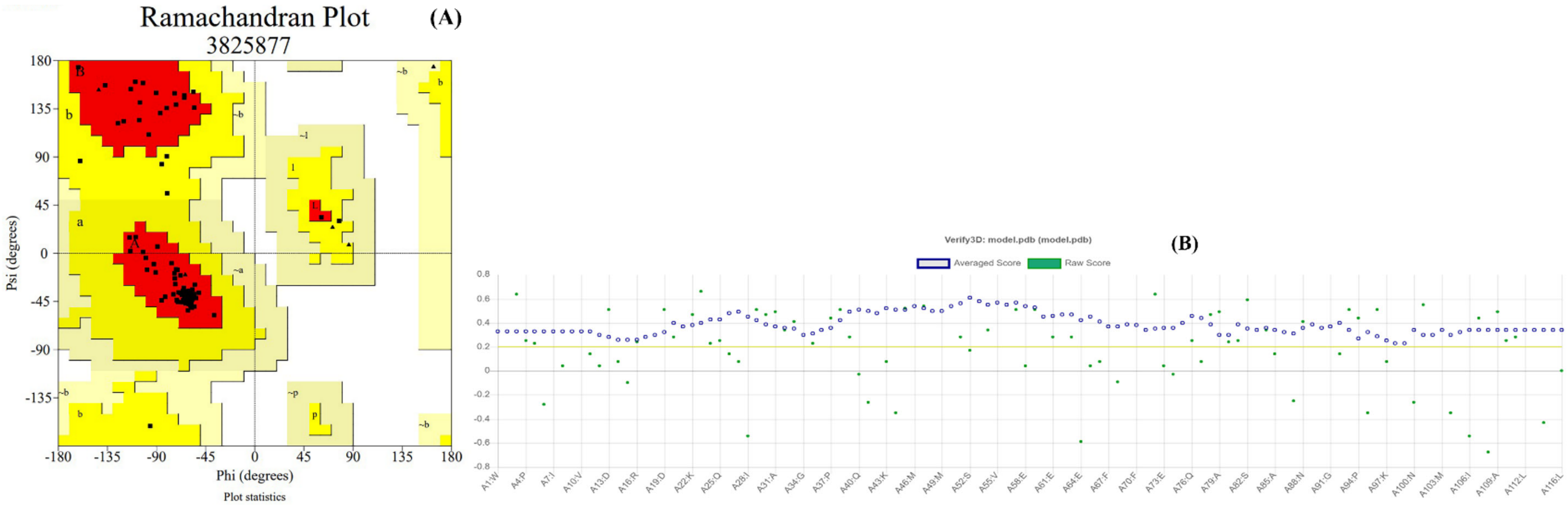

Figure S4. (A): Ramachandran plot and (B): Verify 3D plots of SnocOBP9.

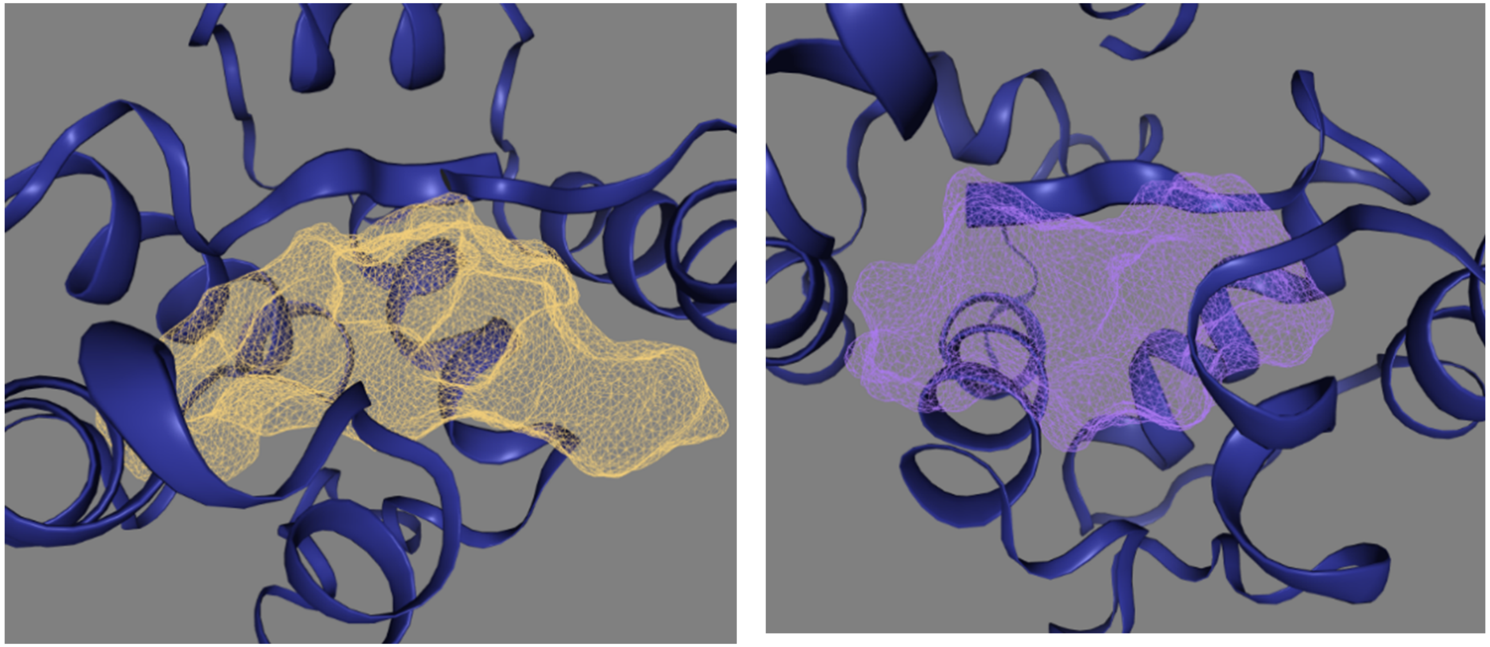

(A) (B)

Figure S5. (A): AmelASP1 binding pocket; (B): SnocOBP9 binding pocket.

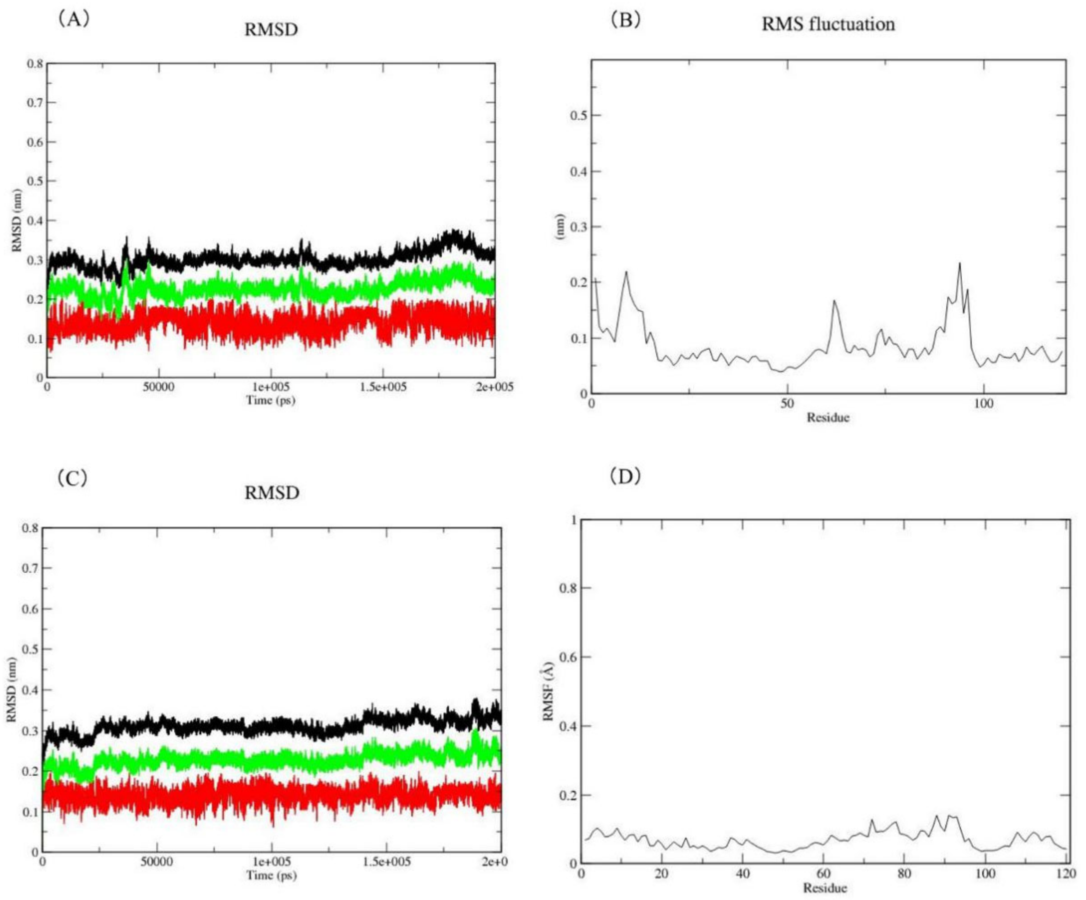

Figure S6. The two repetitions of the 200-ns MD simulations of the SnocOBP9-Z3D complexes. (A) and (C): The time-evolution RMSD curves of SnocOBP9-Z3D complex; (B) and (D): SnocOBP9-Z3D complex RMSF curve.

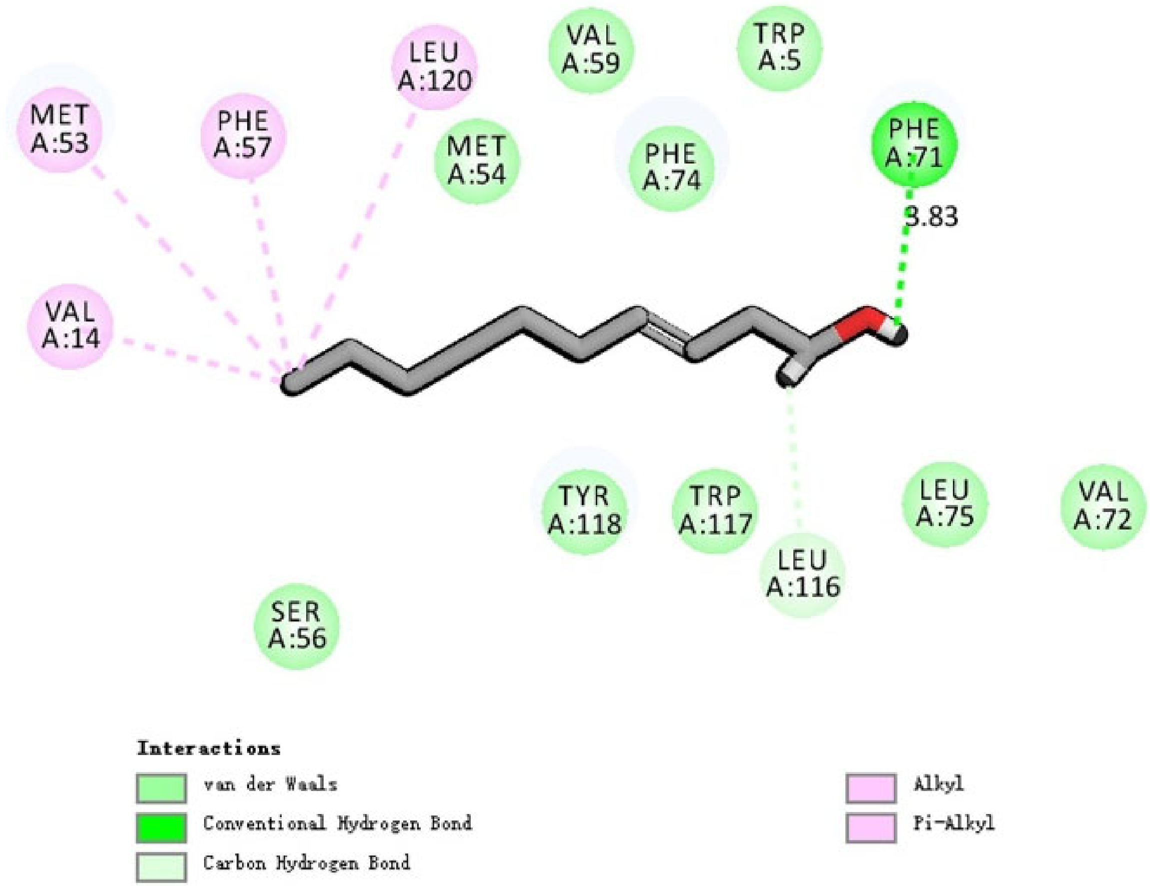

Figure S7. 2D diagram plot of SnocOBP9-Z3D interaction, 3.83 indicates the distance of the hydrogen bond between residue Phe 71 and Z3D.

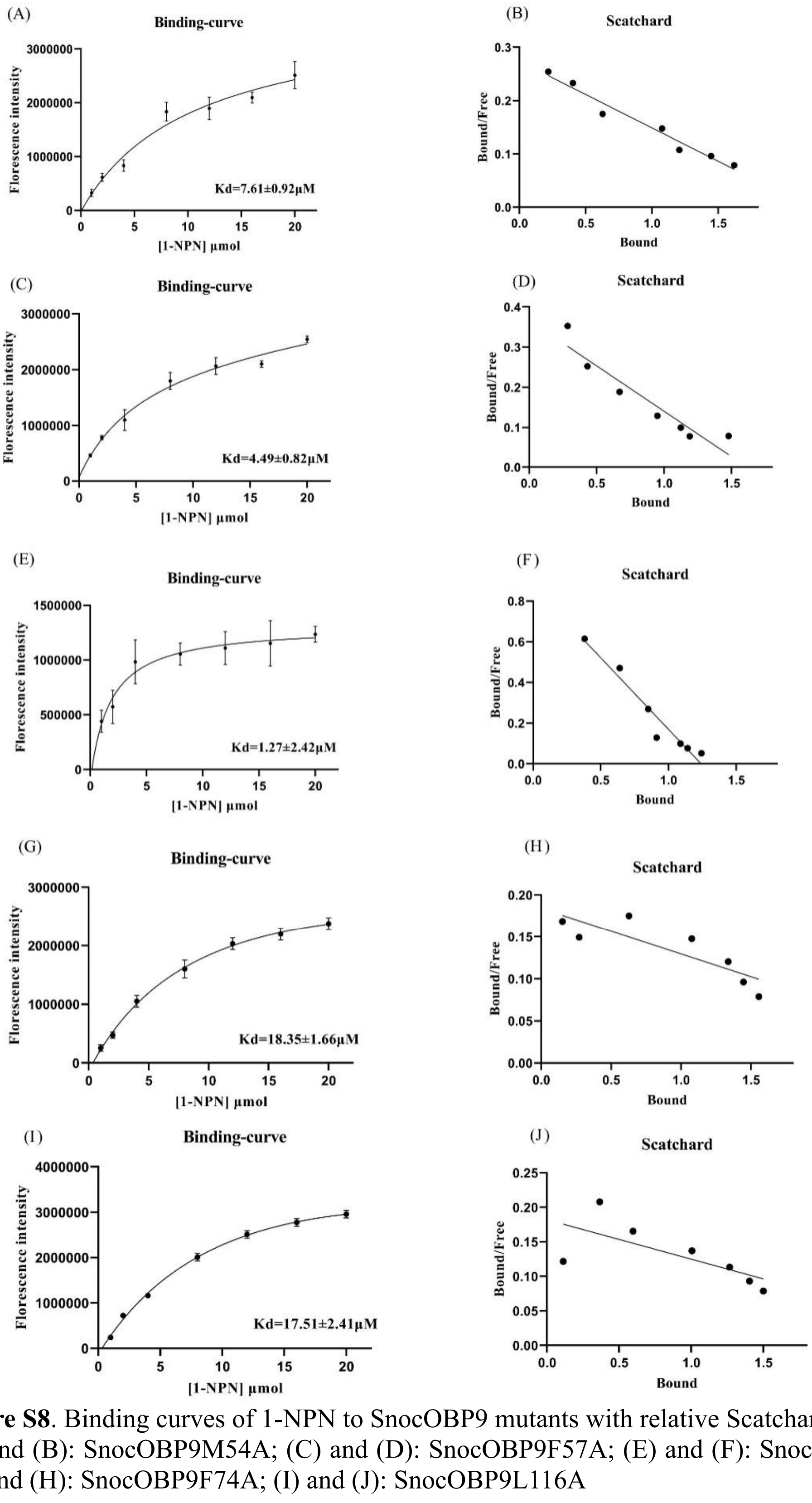

Figure S8. Binding curves of 1-NPN to SnocOBP9 mutants with relative Scatchard plots. (A) and (B): SnocOBP9M54A; (C) and (D): SnocOBP9F57A; (E) and (F): SnocOBP9F71A; (G) and (H): SnocOBP9F74A; (I) and (J): SnocOBP9L116A

Table S1. Autodock Vina score.

Conformation	Binding energy[a]
1	-6.67
2	-6.5
3	-6.48
4	-6.38
5	-6.25
6	-6.15

[a]All energy values are given in kcal/mol

Table S2. The Computational Alanine Scanning (CAS) for the important residues contributing to the binding mode.

Complex	Mutation	State after Mutation	Mutation Energy[a] ($\Delta\Delta G_{mut}$)
SnocOBP9-Z3D	MET54>ALA	DESTABILIZING	0.58
	PHE57>ALA	DESTABILIZING	0.54
	PHE71>ALA	DESTABILIZING	1.18
	PHE74>ALA	DESTABILIZING	1.42
	LEU116>ALA	DESTABILIZING	0.55

[a]All energy values are given in kcal/mol.

Table S3. Renaturation solution type and the renaturation process.

Renaturation solution type	Sodium chloride[a]	Tris-HCl[b]	Urea[a]	Reduced glutathione[b]	Oxidized glutathione[b]	Glycerol	pH	Solvent
Renaturation solution A	0.5	20	8	5	0.5	10%	8.0	Ultrapure water
Renaturation solution B	0.5	20	4	5	0.5	10%	8.0	Ultrapure water
Renaturation solution C	0.5	20	3	5	0.5	10%	8.0	Ultrapure water
Renaturation solution D	0.5	20	2	5	0.5	10%	8.0	Ultrapure water
Renaturation solution E	0.5	20	1	5	0.5	10%	8.0	Ultrapure water
Renaturation solution F	0.5	20	0.5	5	0.5	10%	8.0	Ultrapure water
Renaturation solution G	0.5	20	-	-	-	10%	8.0	Ultrapure water
Renaturation solution H	-	-	-	-	-	-	8.0	PBS[c]

[a] All values are given in mol/L.
[b] All values are given in mmol/L.
[c] Phosphate buffer solution value is given in 0.01 mol/L.
Dilute the protein concentration to less than 1 mg/ml, dialyzed with the renaturation solution, change it every 6 hours, replace from A-H. And finally determine the concentration by BCA, below 1 mg/ml, use the ultrafiltration tube concentration, and finally re-set the concentration, Save at -20 °C.
